# The effect of an anti-malarial herbal remedy, *Maytenus senegalensis*, on electrocardiograms of healthy Tanzanian volunteers

**DOI:** 10.1186/s12936-024-04935-w

**Published:** 2024-04-12

**Authors:** Kamaka R. Kassimu, Ali M. Ali, Justin J. Omolo, Abel Mdemu, Francis Machumi, Billy Ngasala

**Affiliations:** 1 Bagamoyo Clinical Trial Facility, Ifakara Health Institute, 74, Bagamoyo, Tanzania; 2 Traditional Medicine Research and Development Center, National Institute for Medical Research, 9653, Dar es Salaam, Tanzania; 3 Institute of Traditional Medicine, Muhimbili University of Health and Allied Sciences, 65001, Dar es Salaam, Tanzania; 4 Department of Parasitology, Muhimbili University of Health and Allied Sciences, 65001, Dar es Salaam, Tanzania

**Keywords:** Electrocardiographic effects, Herbal remedy, Healthy volunteer, *M*. *senegalensis*

## Abstract

**Background:**

The emergence of resistance to artemisinin-based combination therapy necessitates the search for new, more potent antiplasmodial compounds, including herbal remedies. The whole extract of *Maytenus senegalensis* has been scientifically investigated for potential biological activities both in vitro and in vivo, demonstrating strong antimalarial activity. However, there is a lack of data on the electrocardiographic effects of *M. senegalensis* in humans, which is a crucial aspect in the investigation of malaria treatment. Assessing the electrocardiographic effects of *M. senegalensis* is essential, as many anti-malarial drugs can inadvertently prolong the QT interval on electrocardiograms. Therefore, the study's objective was to evaluate the electrocardiographic effects of *M. senegalensis* in healthy adult volunteers.

**Methods:**

This study is a secondary analysis of an open-label single-arm dose escalation. Twelve healthy eligible Tanzanian males, aged 18 to 45, were enrolled in four study dose groups. A single 12-lead electrocardiogram (ECG) was performed at baseline and on days 3, 7, 14, 28, and 56.

**Results:**

No QTcF adverse events occurred with any drug dose. Only one volunteer who received the highest dose (800 mg) of *M. senegalensis* experienced a moderate transient change (△QTcF > 30 ms; specifically, the value was 37 ms) from baseline on day 28. There was no difference in maximum QTcF and maximum △QTcF between volunteers in all four study dose groups.

**Conclusions:**

A four-day regimen of 800 mg every 8 h of *M. senegalensis* did not impact the electrocardiographic parameters in healthy volunteers. This study suggests that *M. senegalensis* could be a valuable addition to malaria treatment, providing a safer alternative and potentially aiding in the battle against artemisinin-resistant malaria. The results of this study support both the traditional use and the modern therapeutic potential of *M. senegalensis*. They also set the stage for future research involving larger and more diverse populations to explore the safety profile of *M. senegalensis* in different demographic groups. This is especially important considering the potential use of *M. senegalensis* as a therapeutic agent and its widespread utilization as traditional medicine.

*Trial registration* ClinicalTrials.gov, NCT04944966. Registered 30 June 2021-Retrospectively registered, https://clinicaltrials.gov/ct2/show/NCT04944966?term=kamaka&draw=2&rank=1

## Background

Malaria remains a major global public health problem due to its association with high rates of illness and mortality. The 2023 World Malaria Report of the World Health Organization (WHO) stated that 249 million malaria cases occurred worldwide in 2022, resulting in the loss of 608,000 lives [1]. Artemisinin-based combination therapy (ACT) has been the most extensively used and successful control strategy for treating uncomplicated malaria [2]. However, the imminent threat of resistance to artemisinin and partner drugs jeopardizes global progress in controlling and eliminating malaria [3]. Furthermore, partial artemisinin resistance has been confirmed in Africa, specifically in Eritrea [4], Rwanda [5], and Uganda [6]. Therefore, the search for new, more potent antiplasmodial compounds, including herbal remedies, is necessary [7].

The use of herbal remedies for the treatment of malaria has been reported in ancient civilizations. There are more than 1000 species used to treat fevers and malaria [8]. For the first time in 1977, the World Health Assembly (WHA) pointed out the potential efficacy of herbal medicine in the national health systems of member countries [9]. In African endemic countries, herbal medicine has been used as an anti-malarial since ancient times, and without it, malaria would have devastated Africa [10, 11]. Recent estimates show that 80% of the African population uses herbal remedies [12]. Herbs are plants or parts of plants that are used for their taste, fragrance, or medicinal purposes and can be obtained in different forms such as fresh or dried plants, tablets, capsules, powders, teas, and extracts [13]. *Maytenus senegalensis* is one of the medicinal plants belonging to the Celastraceae family. It is a shrub or tree (known under the common name *spike thorn*) that grows up to 15 m high and is widely distributed in most African countries, as well as Arabia, Afghanistan, and India [14].

The whole extract of *M. senegalensis* has been scientifically investigated for potential biological activities both in vitro [15–17] and in vivo [18–20], demonstrating strong anti-malarial activity.

This treatment approach using whole plant extracts is believed to be more appropriate and has higher chances of success. Reports from animal models suggest that extracts from the whole plant *Artemisia annua* have a low tendency to gain parasite resistance compared to the purified product artemisinin [21], which may be due to the synergistic effect of various compounds within the extract.

In Tanzania, *M. senegalensis* is one of the most important medicinal plants for the treatment of malaria, fever, pain, and chronic diseases [18]. Traditional uses of *M. senegalensis* have also been reported in other African countries, such as Benin, Ivory Coast, Kenya, Sudan, Zambia, and Zimbabwe [15, 22, 23].

The history of medicine reminds us that no therapeutic advance comes without vigilance. The discovery of halofantrine’s cardiotoxicity following its registration and introduction into clinical practice has brought attention to the significance of properly evaluating the potential cardiotoxic effects of newly introduced anti-malarial medications [24–26]. The urgent need for this scrutiny is underscored by the recognition that many anti-malarial drugs can inadvertently induce prolongation of the electrocardiographic QT interval [24, 27–31].

The QT interval in the PQRST complex of an ECG, represents the time for ventricular depolarization and repolarization in the cardiomyocytes and is correlated with heart rate; a higher heart rate results in a shorter QT interval and vice versa [32]. Therefore, multiple formulas are used to adjust the interpreted QT interval for heart rate to obtain the corrected QT interval (QTc). Among the frequently employed formulae are those by Bazett (QTcB) and Fridericia (QTcF) [33, 34]. Drugs that cause TdP block the heart’s herG potassium channel, but not all herG blockers lead to TdP [35]. The US Food and Drug Administration has noted that a prolonged QTc interval > 60 ms increases the risk of TdP [36, 37].

In spite of the evaluation of the whole extract of *M. senegalensis* for acute toxicity and antiplasmodial activity, an important area—the electrocardiographic effects of *M. senegalensis* in humans—remains unexplored. Given that myriad factors can affect the QT interval, including malaria infection [28], the deliberate investigation of the electrocardiographic effects of *M. senegalensis* in a cohort of healthy adult volunteers, not only prevents incorrect interpretation of electrocardiographic data but also enhances the accuracy and applicability of our findings.

The objective of this study was to evaluate the electrocardiographic effects of *M. senegalensis* in a group of healthy adult volunteers. As the repercussions of malaria’s impact continue to reverberate, emphasizing the need for novel approaches, this study contributes a vital component to the evolving array of anti-malarial strategies.

## Methods

### Study design and ethical considerations

This study presents a secondary analysis data of clinical trial conducted at the Bagamoyo Clinical Trial Facility (BCTF) from June to September 2021. The trial aimed to assess the safety and tolerability of the anti-malarial herbal remedy *M. senegalensis* in healthy adult Tanzanian volunteers aged 18 to 45 years. The study design has been previously described in detail elsewhere [38]. Briefly, 12 study volunteers were randomized into four groups of three volunteers each and gave their written informed consent to participate. Study group 1 received 400 mg of *M. senegalensis,* study group 2 received 600 mg, and study groups 3 and 4 received 800 mg every 8 h for 4 days.

### Inclusion and exclusion criteria

The inclusion criteria were as follows: (1) Healthy men aged 18 to 45, with a BMI ranging from 18 to 30 kg/m2; (2) Long-term residents of the study area; (3) Willingness to disclose medical information and undergo examinations by a study clinician before and during the study; (4) Willingness to provide contact details of a third-party household member or close friend to the study team; (5) Availability by mobile phone at all times during the study period; (6) Non-participation in any other clinical trial or blood donation during the study period; (7) Adherence to study protocol procedures; (8) Willingness to undergo HIV, hepatitis B (HBV), and hepatitis C (HCV) tests; (9) Demonstrated understanding of the study; (10) Provision of written informed consent; and (11) Absence of malaria parasitaemia, confirmed by blood smear at enrollment.

The exclusion criteria were as follows: (1) Previous use of an investigational malaria drug within the past 5 years; (2) Participation in any other clinical study involving investigational medicinal products within 30 days before the start of this study; (3) History of arrhythmias, prolonged QT interval, or other cardiac disease; (4) Clinically significant abnormalities in the electrocardiogram (ECG) at screening; (5) Positive family history of cardiac disease in a first or second-degree relative at age 50; (6) History of psychiatric disease or suffering from any chronic disease (e.g., diabetes mellitus, cancer, HIV/AIDS); (7) Confirmed or suspected immunosuppressive or immune-deficient condition; (8) History of drug or alcohol abuse; (9) Chronic use of immunosuppressive or other immunomodifying drugs within three months before the study, except for inhaled and topical corticosteroids; (10) Clinically significant deviation from the normal range in biochemistry, hematology, blood tests, or urine analysis; (11) Positive HIV, hepatitis B virus, or hepatitis C virus tests; (12) Suspected clinically active tuberculosis (TB) or positive QuantiFERON-TB Gold Test in-tube assay; (13) Symptoms, physical signs, or laboratory values suggestive of systemic disorders that could affect the interpretation of study results or jeopardize volunteer health; (14) Medical, social, or occupational reasons contraindicating participation, impairing the ability to provide informed consent, increasing risk to the volunteer, affecting participation in the study, or impairing interpretation of study data, as judged by the study clinician.

### Investigation product preparation

The investigational product preparation details have been previously reported [38]. In brief, On June 9, 2020, root barks of *M. senegalensis* were harvested in Bugabo Buzi village, Missenyi District, Kagera Region. The pulverized root barks, totaling 20 kg, were extracted three times with 80% ethanol through cold percolation at room temperature. The resulting filtrates were then passed through Macherey–Nagel filter paper. Subsequently, the filtrates underwent distillation under reduced pressure at 40 ℃, resulting in the production of 2.24 kg of brown gummy extract. The presence of the active ingredient, Pristimerin, was qualitatively determined using the Thin Layer Chromatography (TLC) method. Finally, the extract was granulated by blending it with starch in a 2:1 ratio (2 parts extract to 1 part starch) and encapsulated in hard-shell capsules.

### ECG measurement

The study measured a single 12-lead ECG at various time points, including baseline and 3, 7, 14, 28, and 56 days, using an ECG machine from Nihon Kohden (ECG 1550 K) in Tokyo, Japan. Participants were assessed in a supine position after a brief rest in the same position. To correct for heart rate, the QTcF and QTcB were calculated using Fridericia’s and Bazett’s formulas, respectively:

$$QTcF = Q{T/RR}^{1/3}$$

$$QTcB = {QT/RR}^{1/2}$$

The difference between the QTc values before and after the drug intervention was used to calculate the QTc change. If the QT interval was prolonged by more than 30 ms, it was classified as moderate, and if it was prolonged by more than 60 ms, it was classified as severe [36, 37].

### Statistical analysis

Statistical analysis was done using STATA (version 15; StataCorp, College Station, TX, USA) and R Statistical Software (version 3.4.3, https://www.r-project.org/). Data visualization was performed using R statistical software (version 3.4.3, https://www.r-project.org/). Weight and height measurements were used to calculate the body mass index, and frequency tables were employed to describe distributions of categorical data. For continuous data, median, interquartile range, and 2.5th and 97.5th percentile ranges were calculated. To compare the distributions of the two groups, the Wilcoxon-Mann Whitney test was used.

## Results

### Study volunteers

A total of 30 male volunteers were screened for inclusion in the study and 12 of them were enrolled. Table 1 presents the baseline demographic information of the volunteers. In general, the median age (2.5th, 97.5th range) and the body mass index were 26 (20.1, 41.5) years and 22.2 (18.5, 27.2), respectively. The median dose (2.5th–97.5th range) was 5.1 (4.9–5.7) mg/kg/day for volunteers who received 400 mg, 11.5 (9.6–12.0) mg/kg/day for volunteers who received 600 mg, and 39.3 (34.5–47.2) mg/kg/day for volunteers who received 800 mg.

**Table 1:** Baseline demographic characteristics of study participants

Parameter	Group 1 (N = 3)	Group 2 (N = 3)	Group 3 and 4 (N = 6)
Age (years)	28.2 (24.4, 29.2)	23.7 (19, 28.3)	26 (24.7, 43)
Weight (Kg)	78 (70.4, 81.1)	52 (50.1, 62.5)	61 (51, 69.5)
Height (cm)	169 (166, 174)	164 (163, 168)	169 (161, 173)
BMI (Kg/m^2)	27.1 (25.5, 27.3)	18.6 (18.4, 23.5)	21.9 (19.7, 23.1)

Group 1: Volunteers received 400 mg of *Maytenus senegalensis* once; Group 2: volunteers received 600 mg of *M. senegalensis* once; Group 3 and 4: volunteers received 800 mg of *M. senegalensis* every 8 h for 4 days. Values are reported as median (2.5th, 97.5th) percentile range

### Prolongation of QTcF interval

**Fig. 1:**
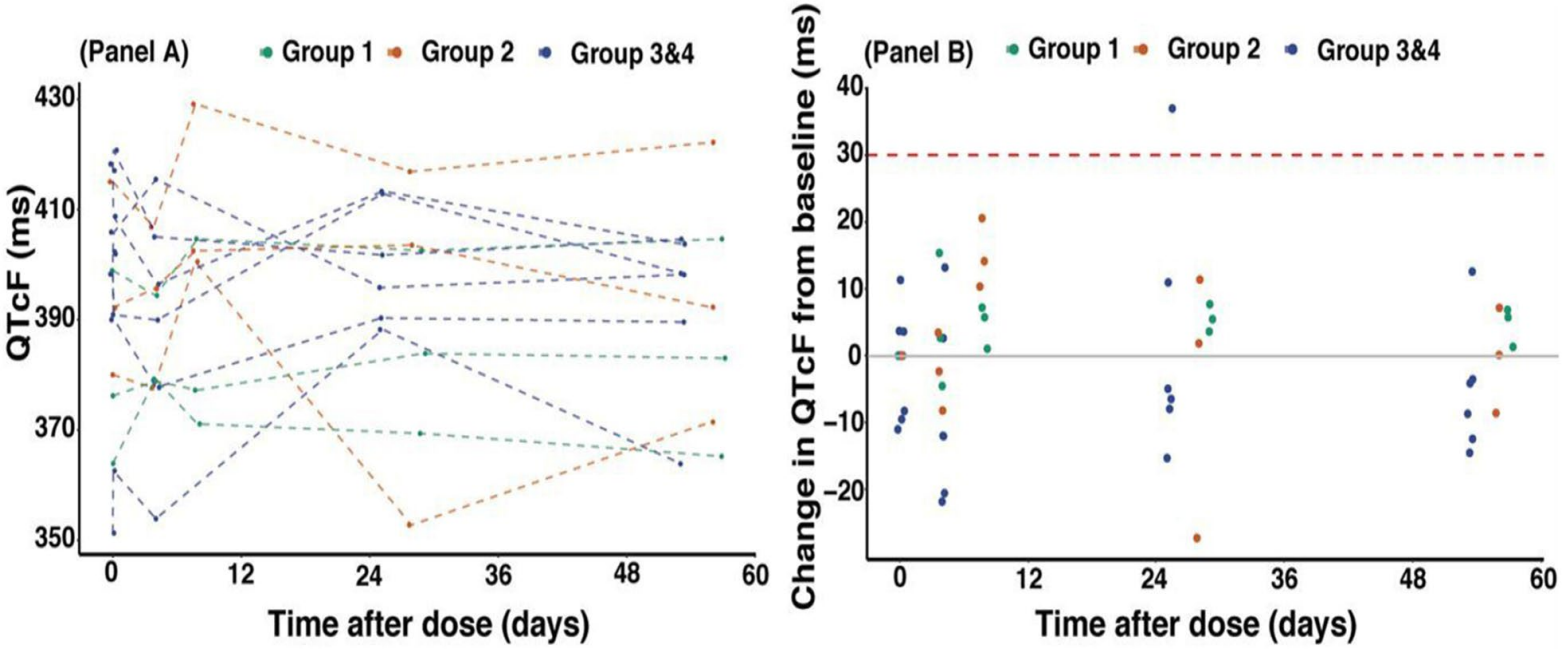
QTcF profiles (**A**) and change in QTcF from predose over time (**B**) in volunteers received *Maytenus senegalensis*. Dashed lines in pane A represent individual volunteers who received 400 mg once (group 1, green line), 600 mg once (group 2, orange line) and 800 mg every 8 h for 4 days (group 3 and 4, blue line). Dots in panel B represent individual observations. QTcF = Fridericia-corrected QT interval. Dashed red line represents a change in QTcF from pre-dose of 30 ms. Solid gray line represents a change in QTcF from pre-dose of 0 ms

Table 2 and Fig. 1A present the electrocardiogram parameter profile of the study participants. All parameters were within the acceptable range (Table 2), although there was notable intra- and interindividual variability in QTcF measures (Fig. 1A). A total of 48 ECG measurements were collected across four visits, and no QTcF adverse events were reported for any dose of *M. senegalensis*. Notably, the highest maximum DQTcF (14.1 ms) was observed in participants who received 600 mg of *M. senegalensis*, compared to those who received 400 mg (7.7 ms) or 800 mg (7.3 ms) (Table 3).

**Table 2:** Electrocardiogram’s parameters in health volunteers receiving *Maytenus senegalensis*

ECG Parameters,	Visit code	Group 1			Group 2			Group 3 and 4		
		n	Values	Change from baseline	n	Values	Change from baseline	n	Values	Change from baseline
**HR (Beats/min)**	Baseline	3	74 (72.1, 84.4)	–	3	67 (60.4, 81.3)	–	6	68.5 (57.4, 86.8)	–
	Day 3	3	79 (64.8, 82.8)	− 6.0 (− 9.8, 10.2)	3	71 (67.2, 75.8)	4.0 (− 5.5, 6.9)	6	58.5 (53.4, 94.8)	− 5.5 (− 14.4, 15.8)
	Day 7	3	78 (76.1, 87.5)	2.0 (− 6.5, 15.3)	3	65 (63.1, 72.6)	− 4.0 (− 8.8, 4.6)	6	66.5 (57.1, 89.1)	− 0.5 (− 10.0, 8.8)
	Day 28	3	67 (67, 67)	− 7.0 (− 17.4, − 5.1)	3	73 (70.2, 75.9)	6.0 (− 11.1, 15.5)	6	66 (58.1, 73.5)	− 3.5 (− 14.1, 1.9)
	Day 56	3	67 (65.1, 75.6)	− 9.0 (− 17.5, 3.4)	3	67 (66, 80.3)	− 1.0 (− 1.0, 6.6)	6	67 (58.1, 94.4)	1.5 (− 9.2, 7.9)
**QTc Fridericia (ms)**	Baseline	3	376.2 (364.5, 397.8)	–	3	392.2 (380.6, 413.9)	–	6	402.1 (357.1, 418.1)	–
	Day 3	3	379.2 (378.9, 393.6)	2.7 (− 4.2, 14.7	3	395.6 (378.5, 406.3)	− 2.4 (− 7.9, 3.1)	6	398.4 (366, 419.2)	− 2.3 (− 10.8, 10.4)
	Day 7	3	377.2 (371.4, 403.3)	5.7 (1.3, 7.1)	3	402.5 (400.6, 427.8)	14.1 (10.5, 20.2)	6	393.2 (356.9, 414.2)	− 12.0 (− 21.7, 11.8)
	Day 28	3	383.9 (370.1, 401.6)	5.5 (3.7, 7.6)	3	403.5 (355.3, 416.2)	1.8 (− 25.8, 10.9)	6	398.8 (388.5, 413.2)	− 5.7 (− 14.4, 33.7)
	Day 56	3	383 (366.1, 403.6)	5.7 (1.5, 6.8)	3	392.3 (372.5, 420.7)	0.1 (− 8.2, 6.8)	6	398.3 (367.1, 404.5)	− 6.4 (− 14.3, 10.6)
QTc Bazett (ms)	Baseline	3	389 (387.1, 411.8)	–	3	400 (383.8, 437)	–	6	413.5 (380.1, 426.2)	–
	Day 3	3	399 (397.1, 400)	10 (− 12.8, 1)	3	409 (388.1, 422.3)	4 (− 15, 8.8)	6	397.5 (389.2, 430.5)	− 10.5 (− 23.2, 18)
	Day 7	3	403 (388.8, 420.1)	8 (1.4, 13.7)	3	407 (406, 442.1)	6 (5.1, 23.1)	6	401.5 (378.4, 431.1)	− 11 (− 30.4, 19)
	Day 28	3	393 (378.8, 411)	− 1 (− 8.6, 3.8)	3	417 (370.4, 428.4)	− 10 (− 14.8, 15.7)	6	406.5 (395.8, 421.8)	− 3 (− 25.4, 25.8)
	Day 56	3	400 (375.3, 410.4)	− 2 (− 12.4, 10.4)	3	400 (380, 443.7)	0 (− 3.8, 6.7)	6	405 (390.5, 414.9)	− 8.5 (− 18.4, 15.9)

Group 1: volunteers received 400 mg of *M. senegalensis once; Group 2: volunteers received 600 mg of M. senegalensis once; Group 3 and 4: volunteers received 800 mg of M.senegalensis every 8 h for 4 days. Values are reported as n or median (2.5th, 97.5th) percentile range*

**Table 3:** Summary of QTcF-prolongation by treatment group

Variable	Group 1	Group 2	Group 3 and 4
ECG measurements	12	12	24
QT prolongation events	0	0	0
Grade 1 (mild)	0	0	0
Grade 2 (moderate)	0	0	0
Grade 3 (severe)	0	0	0
Maximum △QTcF (ms)	7.7 (5.8, 15)	14.1 (11.5, 20.2)	7.3 (− 7.6, 34)
Events with DQTcF > 30 ms < 60 ms	0	0	1
Events with DQTcF ≥ 60 ms	0	0	0
Maximum QTcF (ms)	383.9 (379.5, 403.6)	403.5 (400.7, 427.9)	413.1 (388.5, 420.1)
Time after dose at maximum QTcF (days)	7.9 (4.2, 27.9)	7.7 (7.7, 26.9)	28.9 (4.4, 29.1)

Group 1: volunteers received 400 mg of *Maytenus senegalensis* once; Group 2: volunteers received 600 mg of *M. senegalensis* once; Group 3 & 4: volunteers received 800 mg of *M. senegalensis* every 8 h for 4 days; QTcF: QT interval corrected by Fridericia formula; DQTcF: change in QTcF from baseline; mild QT prolongation: QTcF interval > 450 to ≤ 480 ms; moderate QT prolongation: QTcF interval > 480 to ≤ 500 ms; severe QT prolongation: QTcF interval > 500 ms. Values are reported as n or median (2.5th, 97.5th) percentile range

**Fig. 2:**
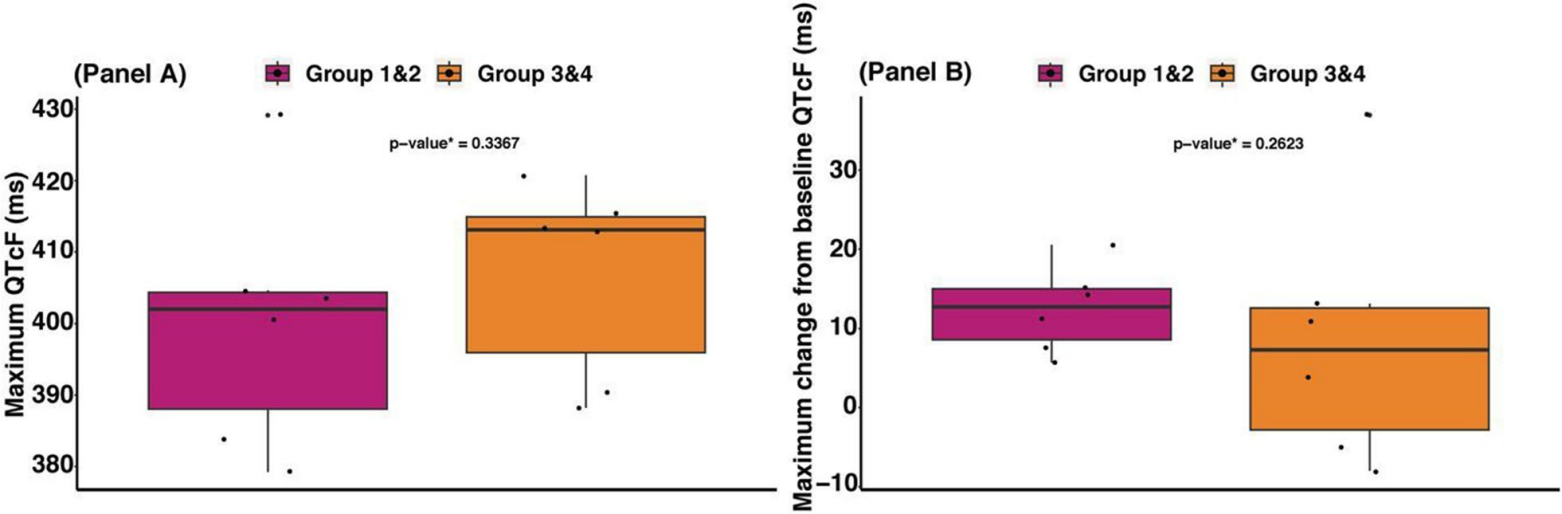
Maximum (**A**) and maximum change (** B**) QTcF (ms) after drug administration. Violet-red colour represents volunteers who received 400 mg and 600 mg once all together (group 1 and 2); Dark-orange color represents volunteers who received 800 mg every 8 h for 4 days (group 3 and 4); *QTcF* QT interval corrected by Frederica formula. Boxplots represent the median, interquartile range, and whiskers show 95th and 5th percentile. *p-value from Wilcoxon-Mann Whitney test

One participant who received 800 mg of *M. senegalensis* every 8 h for 4 days had a maximum △QTcF > 30 ms; this value of 37 ms occurred on day 28 (Table 3 and Fig. 1B). Maximum QTcF and maximum △QTcF did not differ significantly between participants who received 800 mg of *M. senegalensis* every 8 h for 4 days and those who received 400 mg and 600 mg combined (Fig. 2).

## Discussion

The primary objective of this study was to assess the electrocardiographic effects of *M. senegalensis* in a cohort of 12 healthy adult volunteers in Tanzania. The study evaluated the electrocardiographic parameters of the participants after controlled exposure to *M. senegalensis* to achieve this goal. In light of the study's research objective, significant insights have been generated that shed light on the potential implications and applications of *M. senegalensis* in the context of cardiac health. This study represents the first investigation into the electrocardiographic effects of *M. senegalensis* in humans. The findings, derived from a cohort of 12 healthy individuals, demonstrate that various dosage regimens of *M. senegalensis* extract did not induce a significant prolongation of the QT interval. These results underscore the favorable cardiac profile of *M. senegalensis*, enhancing its overall safety profile.

A change of > 30 ms (moderate) in the QT interval from the baseline may raise concern, and a change of > 60 ms (severe) may raise even greater concern regarding the potential for arrhythmias [36, 37]. According to the correction of the QT interval made by the Federica formula, only one volunteer who received the highest dose in this study had a moderate transient change from baseline on day 28. As there have been no studies on drug metabolism, it is unclear whether the abnormality observed in one patient after 28 days was caused by the trial or by some other factor.

The study provides critical safety information regarding use of *M. senegalensis*. The absence of significant electrocardiographic effects in healthy volunteers is reassuring from a cardiac safety standpoint. The findings suggest that, *M. senegalensis* is less likely to elicit the cardiac arrhythmias associated with QT interval prolongation within the studied dose range and regimen. This favourable safety profile enhances the prospects of *M. senegalensis* as a potential therapeutic agent, particularly compared to other antimalarial drugs that carry cardiotoxic risks. From the perspective of traditional use, the findings support the traditional wisdom surrounding *M. senegalensis* as an herbal remedy for malaria and other conditions.

Torsades de pointes often occur in the presence of multiple risk factors, including QTc prolongation, female sex, advanced age (> 65 years), bradycardia, hypokalemia, and underlying heart disease [37, 39–43].

It is worth noting that the QT interval begins to exhibit sex-based differences during puberty due to the influence of sex hormones, making female sex a known risk factor for QT prolongation [39]. In addition, the literature has shown that malaria infection can affect the QT interval. Fever, which often accompanies malaria, can lead to an increase in core temperature. The increase in core body temperature by 1 ℃ may result in an 8.5-beat-per-minute (bpm) increase in heart rate of 8.5 beats per minute (bpm). These heart rate changes are closely related to alterations in the QTc interval [31]. The study cohort consisted of healthy adult male volunteers aged 18 to 45 years. This composition effectively neutralizes many of the aforementioned risk factors, thereby adding a substantial layer of confidence to the safety profile of *M. senegalensis*.

While recognizing the significant progress the study has made, it is essential to take into account its limitations. The first limitation is the enrollment of a small number of male volunteers. It is clear that this calls for caution when extrapolating the study findings to larger populations. However, the data reported here provide a foundation for further clinical research on *M. senegalensis*. The second limitation is that the assignment was not designed with a placebo or blank control group. The absence of a placebo or blank control group in the study design may hinder the ability to accurately assess the true impact of *Maytenus senegalensis* on electrocardiograms of healthy Tanzanian volunteers.

Further, the absence of pharmacokinetic information on *M. senegalensis* extract limited the ability to relate plasma concentrations of its components to the results on the electrocardiogram, which opens a key area for further investigation.

## Conclusions

A dose of 800 mg every 8 h for four days of *M. senegalensis* did not affect the electrocardiographic parameters in healthy volunteers. These findings have broad implications, reaching beyond potential therapeutic applications of *M. senegalensis* to its traditional use as an herbal remedy. Across various cultures, *M. senegalensis* has traditionally treated a range of ailments, including malaria. The results of this study lend support to both its traditional use and its modern therapeutic potential. Furthermore, this study represents a significant step in understanding of the safety profile of *M. senegalensis*, reaffirming its historical significance and bridging the gap between traditional knowledge and modern scientific inquiry.

Moreover, this study on the evaluation of electrocardiographic effects provides a foundation for future clinical research involving larger and more diverse populations. A larger sample size and a potentially broader age range could provide additional information on the safety profile of *M. senegalensis* in different demographic groups. Additionally, the integration of pharmacokinetic evaluation in these studies could provide a more comprehensive understanding of the relationship between plasma concentrations of *M. senegalensis* compounds and their cardiac effects.

The study’s findings also open avenues for the strategic integration of *M. senegalensis* into malaria treatment regimens. *M. senegalensis* could be explored as a potential anti-malarial therapy in the pipeline. The observed lack of QT interval prolongation suggests that *M. senegalensis* might offer a safer option for malaria treatment, addressing the critical need to mitigate cardiotoxic risks associated with some anti-malarial agents.

## Data Availability

The datasets analyzed during the current study are available. Researchers interested in accessing the data may request anonymized data through the Chairperson of the National Health Research Ethics Review Committee. The contact information for submitting data requests is as follows: National Institute for Medical Research (NIMR), 2448 Ocean Road, P.O. Box 9653, Dar es Salaam, Tanzania. Telephone: + 255 22 212,140. Email: ethics@nimr.or.tz.

## Abbreviations

ACT: Artemisinin-based Combination Therapy
AIDS: Acquired Immune Deficiency Syndrome
BCTF: Bagamoyo Clinical Trial Facility
BMI: Body mass index
ECG: Electrocardiogram
HBV: Hepatitis B Virus
HCV: Hepatitis C Virus
HIV: Human Immunodeficiency Virus
IRBs: Institutional Review Boards
NIMR: National Institute of Medical Research
TdP: Torsade de Pointes
TLC: Thin Layer Chromatography
TMDA: Tanzania Medicines and Medical Devices Authority
WHO: World Health Organization
